# Large-scale analysis of *Macaca fascicularis *transcripts and inference of genetic divergence between *M. fascicularis *and *M. mulatta*

**DOI:** 10.1186/1471-2164-9-90

**Published:** 2008-02-24

**Authors:** Naoki Osada, Katsuyuki Hashimoto, Yosuke Kameoka, Makoto Hirata, Reiko Tanuma, Yasuhiro Uno, Itsuro Inoue, Munetomo Hida, Yutaka Suzuki, Sumio Sugano, Keiji Terao, Jun Kusuda, Ichiro Takahashi

**Affiliations:** 1 Department of Biomedical Resources, National Institute of Biomedical Innovation, Ibaraki, Japan; 2 Pharmacokinetics and Bioanalysis Center, Shin Nippon Biomedical Laboratories, Ltd., Kainain, Japan; 3 Division of Genetic Diagnosis, Institute of Medical Science, University of Tokyo, Tokyo, Japan; 4 International Research and Educational Institute for Integrated Medical Sciences, Tokyo Women's Medical University, Tokyo, Japan; 5 Department of Medical Genome Sciences, Graduate School of Frontier Sciences, University of Tokyo, Tokyo, Japan; 6 Tsukuba Primate Center for Medical Science, National Institute of Biomedical Innovation, Tsukuba, Japan

## Abstract

**Background:**

Cynomolgus macaques (*Macaca fascicularis*) are widely used as experimental animals in biomedical research and are closely related to other laboratory macaques, such as rhesus macaques (*M. mulatta*). We isolated 85,721 clones and determined 9407 full-insert sequences from cynomolgus monkey brain, testis, and liver. These sequences were annotated based on homology to human genes and stored in a database, QFbase .

**Results:**

We found that 1024 transcripts did not represent any public human cDNA sequence and examined their expression using *M. fascicularis *oligonucleotide microarrays. Significant expression was detected for 544 (51%) of the unidentified transcripts. Moreover, we identified 226 genes containing exon alterations in the untranslated regions of the macaque transcripts, despite the highly conserved structure of the coding regions. Considering the polymorphism in the common ancestor of cynomolgus and rhesus macaques and the rate of PCR errors, the divergence time between the two species was estimated to be around 0.9 million years ago.

**Conclusion:**

Transcript data from Old World monkeys provide a means not only to determine the evolutionary difference between human and non-human primates but also to unveil hidden transcripts in the human genome. Increasing the genomic resources and information of macaque monkeys will greatly contribute to the development of evolutionary biology and biomedical sciences.

## Background

Genomic resources and information about primates are valuable for evolutionary and biomedical studies to determine how and why phenotypes specific to humans, as well as human diseases, have been formed. Moreover, they are important for extrapolating the results of laboratory experiments to medical research because the physiology of primates is more similar to that of humans as compared with other common experimental animals such as rodents. The cynomolgus macaque (*Macaca fascicularis*), also known as the long-tailed or crab-eating macaque, is an Old World monkey living in Southeast Asia. It is bred in laboratories worldwide and is one of the most popular primates used for laboratory animal studies, such as those on infectious diseases, immunology, pharmacology, tissue engineering, gene therapy, senescence, and learning [1]. Cynomolgus macaques, rhesus macaques (*M. mulatta*), and Japanese macaques (*M. fuscata*) are widely used for experimental studies and are closely related to each other [2-4]. The US government funded genome sequencing of the rhesus macaque because it is the most common laboratory animal bred in the US, and in 2007, the draft sequence of the rhesus macaque was published [5].

Since cynomolgus and rhesus monkeys are very closely related at the genetic level, we aim to determine the extent to which the rhesus macaque genome sequence can be used as a reference for biomedical studies involving cynomolgus macaques. At the chromosomal level, a previous study suggested that a pericentric chromosome inversion occurred in the cynomolgus lineage after splitting from rhesus macaques [6]. At the nucleotide sequence level, the genetic divergence between cynomolgus and rhesus monkeys has been measured using mitochondrial DNA sequences [2,3] or a limited number of loci on the chromosomes [4,7]. Thus, the divergence of a sufficient number of loci between cynomolgus and rhesus macaques would assist in determining the degree of genetic divergence between them. In addition, recent studies have shown that there is a considerable amount of genetic diversity within the species themselves [5-10], which also hampers the measurement of the genetic divergence. Because the divergence between the two macaques is very recent (much later than the divergence between humans and chimpanzees), we must consider the segregation of polymorphisms in the common ancestral population to estimate the correct species divergence time [11,12]. By analyzing the number of loci in the two species, we can determine the history of divergence between them, including the ancestral population size, divergence time between species, and possible gene flow [13,14].

We have constructed full-length-enriched cDNA libraries from cynomolgus monkey brain, testis, and liver using the oligo-capping method. Many comparative genomics projects have focused on sequencing of the genome or expressed sequenced tags (ESTs), and full-length cDNA sequences are uniquely informative resources for accurately predicting the full structure of transcripts in the genome [15]. Furthermore, because cynomolgus and rhesus macaques are very closely related, transcriptome data from cynomolgus macaques is useful for annotating the genome sequence of other macaques whose transcriptome data is less than 1% of that from humans and whose full-length cDNA data is scarce.

Along with the cynomolgus macaque cDNA sequencing project, we have published a part of our results, such as novel gene findings [16-19], search for fast-evolving genes [19], molecular evolution of 5'-untranslated regions (UTRs) [20], and evolution of brain-expressed genes [21]. In this study, we summarize the final sequencing project and present novel findings with an expanded dataset. In total, 85,721 ESTs and 9407 full-length sequences were determined, annotated, and stored in an in-house database and the public databases (DDBJ/EMBL/GenBank). Our study focused on the divergence between the cynomolgus and rhesus macaque genes. We did not intensively analyze the divergence between humans and cynomolgus monkeys, because a study on rhesus genome has investigated this thoroughly [5]; it also identified and discussed positively selected genes or extensively duplicated genomic regions during the evolution of *Catarrhine *primates.

## Results

### Summary of cDNA sequences

We constructed several oligo-capped cDNA libraries from cynomolgus monkey testis, liver, and seven anatomical parts of the brain (cerebellar cortex, parietal lobe, occipital lobe, frontal lobe, temporal lobe, medulla oblongata, and brain stem). The oligo-capping method selectively amplifies full-length cDNAs with a cap structure and poly(A) tail [22]. We sequenced the 5'- or 3'-end of 85,721 clones, yielding 63,395 and 22,326 sequences of 5'- and 3'-ESTs, respectively, after filtering the vector and low quality sequences. These EST sequences grouped into 16,466 clusters with 11,016 singletons (BLAST e-value: 1e-30). We classified them based on homology to the 26,575 non-redundant human RefSeq sequences (see methods). Of the 85,721 EST sequences, 68,257 (80%) were homologous to the human RefSeq gene set and were clustered into 9065 types of genes, indicating that our EST sequences would cover about 34% of the known human transcripts (Table 1). In particular, when we limited the human reference genes to the validated protein-coding genes (*i.e*., RefSeq accession beginning with NM), 47% of the human reference genes were represented in the macaque cDNAs.

**Table 1:** Summary of cDNA clones

**Library**	**# of isolated clones**	**# of full-sequenced clones**
Brain: Parietal Lobe (QnpA)	8063 (5890)	649 (336)
Brain: Frontal Lobe (QflA)	13,215 (9286)	2493 (1768)
Brain: Temporal Lobe (QtrA)	6797 (6039)	1078 (862)
Brain: Occipital Lobe (QorA)	5458 (4518)	634 (606)
Brain Stem (QbsA, B)	2776 (1993)	359 (301)
Brain: Medulla Oblongata (QmoA)	4485 (3645)	1146 (912)
Brain: Cerebellar Cortex (QccE)	11,734 (9028)	731 (563)
Testis (QtsA)	10,867 (8510)	2316 (2175)
Liver (Qlv)	22,326 (20,833)	0 (0)
		
Total	85,721 (69,742)	9407 (7523)
Averaged Length		1882 bp

*Numbers of genes with the RefSeq homologs are shown in parentheses.

In parallel to EST sequencing, we determined about 9500 full-insert sequences of the cDNA clones. About 2500 clones whose 5'-EST sequences were not homologous to the public cDNA sequences and 7000 clones whose 5'-EST sequences were homologous to the human RefSeq sequences were chosen [16-21]. Out of the 9407 full-insert sequences, 7407 sequences were homologous to 5384 types of human genes (Table 1). The averaged length of the full-insert sequences was 1864 bp, excluding the length of the poly(A) tail. The macaque sequences were annotated for gene function and homologous locus in the human genome using information from the Entrez Gene [23] and Gene Ontology (GO) databases [24].

### Database construction

All cDNA sequences and annotations were deposited in the public databases and stored in a simple in-house database, QFbase [25]. On the QFbase website, users can search the macaque clones by keywords and BLAST searches. For each human gene, the distribution of the macaque homologs is represented graphically and users can easily retrieve information of the objective macaque cDNA clones. The entries are further linked to the gene annotation in the outside databases, GenBank [26], Ensembl [27], OMIM [28], and H-InvDB [29]. The cDNA sequences were mapped on the human and rhesus genome sequences using the UCSC genome browser [30]. Moreover, 4665 human-macaque orthologous alignments are provided in the QFbase. For each alignment, the non-synonymous substitution rate (*K*_*a*_) and the synonymous substitution rate (*K*_*s*_) between the human and macaque cDNA sequences were estimated. Non-synonymous substitutions are nucleotide changes that replace amino acids between species whereas synonymous substitutions cause no amino acid changes. The relative pace of protein evolution was thus determined using *K*_*a*_/*K*_*s*_, assuming that the *K*_*s *_value reflects the neutral mutation rate [31]. Using the database, users can sort the alignments according to the *K*_*a *_and *K*_*s *_values. For example, users can determine the *K*_*a *_and *K*_*s *_values of a particular gene or view the list of the 100 most rapidly evolved genes between humans and cynomolgus monkeys. The cDNA clones are distributed through the Human Science Research Resource Bank in Japan (Tokyo, Japan). Further information is available at the QFbase website.

### Analysis of unidentified transcripts

**Figure 1:**
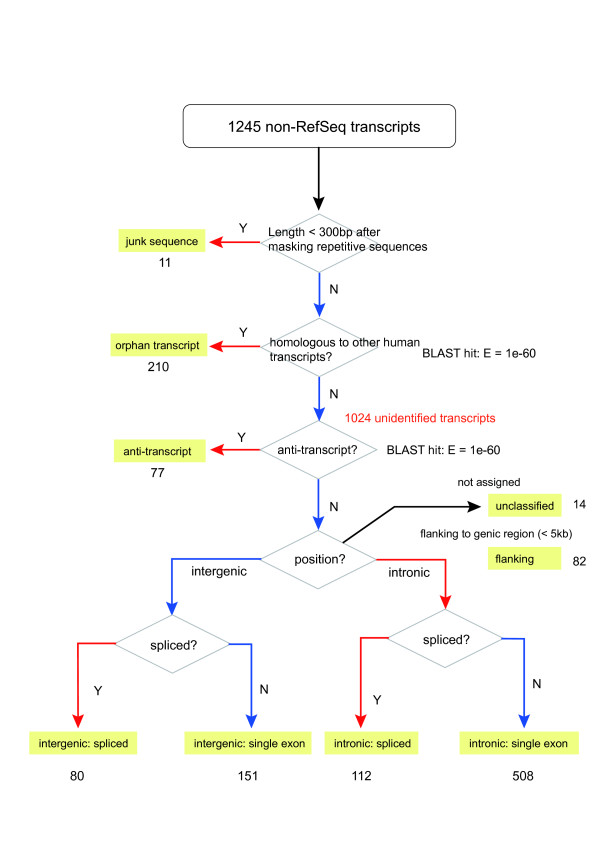
Classification of the 1245 Non-RefSeq transcripts. Transcripts shorter than 300 bp after masking the repetitive sequences were categorized as junk sequences. The remaining sequences were BLAST-searched against all public human cDNA sequences for the forward strand. Homologous sequences to the unannotated human cDNAs were classified as orphan transcripts for the forward strand and anti-transcript for the reverse strand. The remaining 947 clones were mapped on the human genome sequence and arranged according to the annotation from the UCSC genome browser (hg18). The transcripts that overlapped with the genic regions including UTR were classified as intronic transcripts, and the transcripts that were mapped more than 5 kb away from the genic region were classified as intergenic transcripts.

Of the 9407 full-sequenced cDNAs, about 2000 were not homologous to the human reference gene sequences (RefSeq, built on Sep 14, 2006; BLAST: E = 1e-60). These Non-RefSeq transcripts clustered into 1245 non-redundant transcripts, which were further classified as shown in Figure 1. The list of the Non-RefSeq transcripts is provided in Additional file 1. We filtered 11 junk sequences and 210 known transcripts. The 210 transcripts matched with the unannotated human cDNA sequences in the database and were called as orphan transcripts. These may help in further annotation of the human genome.

After removing the junk sequences and orphan transcripts, the remaining 1024 transcripts were referred as the unidentified transcripts although 40% (406/1024) of the transcripts showed homology to human ESTs (BLAST: E = 1e-60), because no full cDNA sequence of humans has been registered in the public databases. One of the advantages of full-length cDNAs is that we can determine the splicing pattern and reading direction of the transcripts in the genome. We categorized the unidentified transcripts as anti-transcript, intronic spliced transcript, intronic single-exon transcript, intergenic spliced transcript, or intergenic single-exon transcript. Among the intergenic transcripts, 82 were located within 5 kb of the genic regions with the same direction as the genes. Of these, 6 were mapped on the upstream regions and 76 were mapped on the downstream regions of the known genes. The result showed they may be hidden extensions of the known transcripts, using alternative promoters and/or poly(A) signals in the human genome. These sequences were filtered from the intergenic transcripts and classified as 'flanking' to genic regions. The largest group was the intronic single-exon transcripts. Although they might be acquired from premature mRNA molecules in the cell nucleus, recent studies have revealed the potential abundance of short intronic transcripts in the human genome [32]. Among these classes, anti-transcripts and intergenic spliced transcripts are the most biologically relevant classes, which are unlikely to be derived from contamination by premature mRNAs.

**Figure 2:**
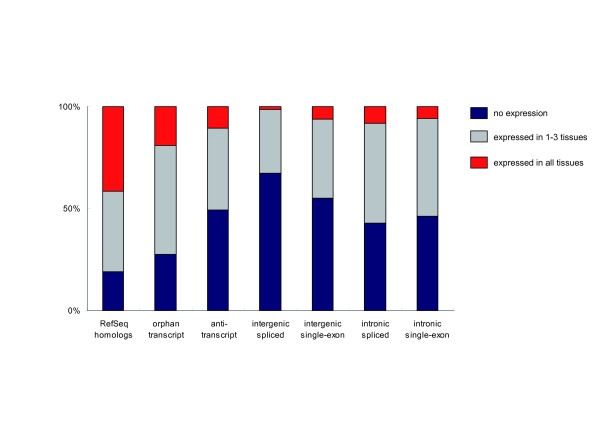
The proportion of the expressed transcripts in the RefSeq homologs (control) and unidentified transcripts. Cerebrum, cerebellum, liver, and testis of a male macaque were used for the microarray experiments with duplicated hybridizations. The transcripts were classified into no expression (blue), expressed in 1–3 tissues (grey), or expressed in all tissues (red).

We designed oligonucleotide microarrays (Affymetrix GeneChip) containing probes complementary to the known genes and unidentified transcripts. Hybridizations were performed using the RNA sampled from a 3-year-old macaque cerebrum, cerebellum, liver, and testis with duplications. The significance of expression was determined using Affymetrix MAS5.0 software [33] (see methods). The proportion of the expressed transcripts is presented in Figure 2. In the unidentified transcripts, 544 transcripts were expressed in at least one of the four tissues (*P *< 0.05; Table 2). Because all the unidentified transcripts were isolated from the macaque brain or testis, fewer transcripts were expressed in the liver (14%) than in the cerebrum (31%), cerebellum (41%), and testis (24%). The expressed proportion of the unidentified transcripts was significantly smaller than that of 8428 RefSeq homologs (51% and 81%, respectively; *P *< 10^-15^; Fisher's exact test). The orphan transcripts were expressed in an intermediate proportion (72%). The percentages of the expressed unidentified transcripts ranged from 33% to 57% (Fig. 1). A large difference was observed between the intergenic and intronic transcripts; more intronic transcripts displayed significant expression on the microarrays than intergenic transcripts (*P *= 0.0005; Fisher's exact test).

**Table 2:** Number of expressed transcripts in the unknown macaque transcripts

	**Unidentified transcripts^a^**	**Intergenic transcripts^b^**
Cerebrum	321	54
Cerebellum	417	58
Liver	139	13
Testis	241	52
All tissues	74	10
Any tissue	544	137

Total	1024	231

^a^Transcripts that have no homology to the public human cDNA sequences.

^b^Transcripts that were mapped more than 5 kb away from the annotated genic regions on the human genome (see Fig. 1).

**Figure 3:**
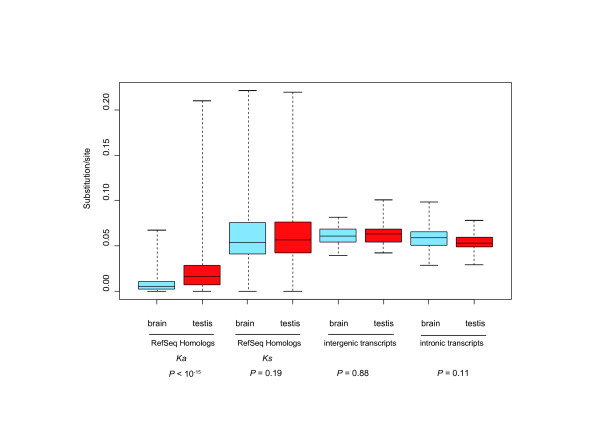
Sequence conservation of the brain-expressed and testis-expressed transcripts between humans and macaques. For the RefSeq homologs (control), the non-synonymous (*K*_*a*_) and synonymous (*K*_*s*_) substitution rates were estimated using the Li-Pamilo-Bianchi method [48]. The substitution rates in the intergenic and intronic transcripts were estimated using Kimura's two parameter methods [55]. The heights of the boxes represent the lower and upper quartile points, and the whiskers show the minimum and maximum points.

Previous studies have shown that many unannotated transcripts are not conserved at a DNA sequence level in many organisms [34]. In practice, sequence conservation is determined by investigating whether the region is alignable. Here, we directly measure the difference in the DNA sequences between humans and macaques. For protein-coding genes, previous studies have shown large disparities in sequence divergence between brain- and testis-expressed genes, both in the CDS and UTR, owing to the stronger functional constraint on the brain-expressed genes [20,21]. We further inquired whether the trend was observed in the unidentified transcripts. We classified the transcripts into brain-expressed transcripts (expressed in the cerebrum and not in the testis) and testis-expressed transcripts (expressed in the testis and not in the cerebrum). As shown in Figure 3, while the non-synonymous substitution rates of the RefSeq homologs were higher in the testis than in the brain, the DNA sequence divergence of the unidentified transcripts was not associated with the expression pattern. Furthermore, there was no evidence that the unidentified transcripts were more conserved than the synonymous sites of the RefSeq homologs.

**Figure 4:**
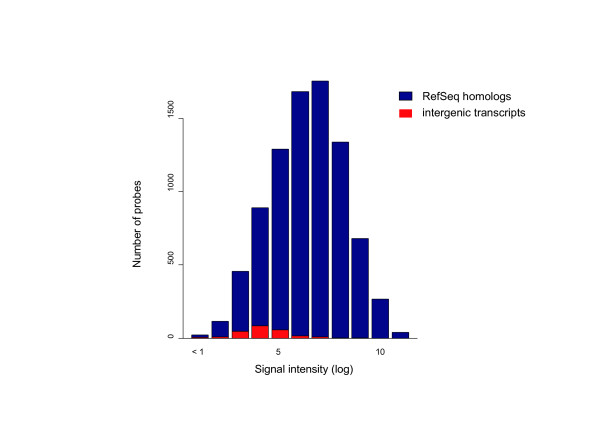
Distribution of transcript expression levels of the RefSeq homologs (blue) and the intergenic transcripts (red). Only the transcripts that were determined as significantly expressed on the microarray are presented in the figure. Log-transformed signal intensity in the tissue with the highest expression was shown. The intergenic transcripts showed significantly lower expression levels than the RefSeq homologs.

**Figure 5:**
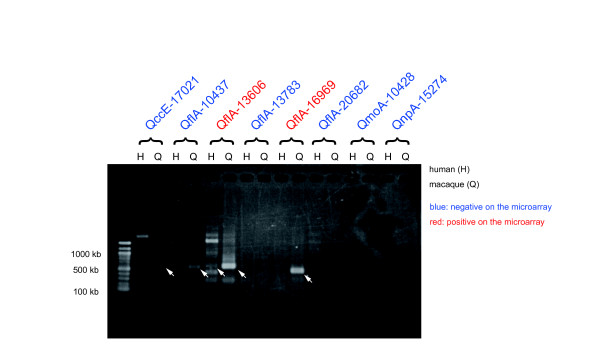
RT-PCR gel images for the expression of the intergenic transcripts in the human (H) and the macaque (Q) brain. Transcript names indicate whether the expression was detected by the microarray experiments (red) or not (blue). Expected PCR products are marked by the white arrows.

We further evaluated the expression level of the 231 intergenic transcripts. We collected the strongest signal intensity of the significantly expressed intergenic transcripts. As shown in Figure 4, even if they were significantly expressed, signal intensities of the intergenic transcripts were significantly weaker than those of the control genes (*P *< 10^-13^; Wilcoxon test). Weak expression levels of intergenic sequences have been previously reported [35,36] and these may cause weak detection levels of the intergenic transcripts. To test the reproducibility of the microarray experiments using another method, we selected eight intergenic spliced transcripts and tried to amplify human and macaque transcripts using RT-PCR. We designed the PCR primers that would match both human and macaque sequences and would amplify introns of genomic sequences when the genomic DNA is contaminated. A gel picture of the RT-PCR products is shown in Figure 5. Two transcripts showed positive results, while six showed negative results on the microarray. We confirmed the expression of the two transcripts in the macaque brain using both the microarray and RT-PCR. Furthermore, even though we failed to detect the expression of the six transcripts on the arrays, we recovered the expression of the two transcripts by RT-PCR. In these two transcripts, the expression levels detected by RT-PCR resulted in considerably weaker bands on the gel (Fig. 5), indicating that the microarray failed to capture their expression at a very low level. In total, we detected the expression of four transcripts in the macaque brain. Of these four transcripts, two were not detected and one was transcribed in an unspliced form in humans. The other showed multiple extra bands in both humans and macaques. Overall, the expression of the macaque intergenic spliced transcripts was not well conserved between the human and the macaque brain.

### Hidden transcript structures in the human genome

**Figure 6:**
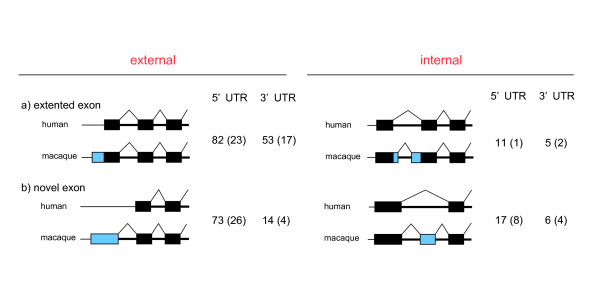
Pattern of the unidentified exons. The closed boxes represent exons in the genomes. Unidentified exons in macaques are presented as blue boxes. Intergenic regions and introns are depicted by thick and thin horizontal lines, respectively. (A) extended exons. (B) novel exons. These exons were further classified into internal (right panel) and external (left panel) exons. The number of genes in each category is shown on the left of each schema. The number of unidentified exons that have not been found even in the EST sequences is shown in parentheses.

Of the 9407 macaque cDNA sequences, 2261 covered the entire CDS of the human RefSeq genes in a single BLAST hit chain. In the 2261 cDNAs, we sought a stretch of UTR sequences (> 50 bp) that did not match any homologous human cDNA sequence. Simple genomic insertion or deletion in the genome was not counted. After filtering the ambiguous entries, in the UTR of macaque cDNAs, we found 262 exons that were not found in the human cDNA data. Out of the 262 unidentified exons, 85 (32%) did not match any human EST sequence. We classified the unidentified UTRs as follows: (A) extended exons and (B) novel exons (Fig. 6). Those unidentified exons were further classified into internal and external exons (Fig. 6). As shown in Figure 1, the distribution of the different types of unidentified exons was not uniform; most of them were external exons.

Because the human transcriptome data is more complex than previously thought, as revealed by genome tiling DNA microarrays [34-36], these unrepresented exons may be expressed at a very low level in human tissues. Moreover, these exons have not been found in the conventional cDNA exploration methods. However, previous studies have suggested a frequent evolutionary turnover of exon sequences [37]. The evolutionary alteration of external exons in the 5'-UTR may be caused by the alternative usage of promoter sequences [38]. The evolutionarily altered exons in the 3'-UTR may be caused by the alternative usage of poly(A) adenylation signals [39]. All the unidentified exons are provided in Additional file 2.

### Comparison of the human, cynomolgus, and rhesus genes

**Figure 7:**
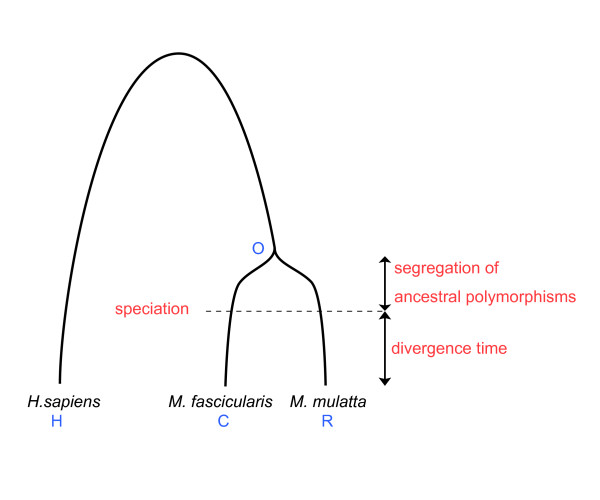
Genealogical relationship (phylogeny of genes) among the humans (H), cynomolgus macaque (C), and rhesus macaque (R). The common ancestor of the two macaques is indicated by the letter O. The time of speciation between the two macaques is shown by the dashed line. Note that the tree is unrooted.

We compiled 2655 human-rhesus-cynomolgus cDNA alignments (dataset I) using the rhesus macaque genome and the predicted transcript sequences. The phylogenetic relationship among the three species is shown in Figure 7. Because the rhesus and cynomolgus genomes are very similar, we wanted to minimize non-orthologous alignments, which inflate the average and variance of the nucleotide divergence between them. Therefore, the macaque genes showing > 80% homology to more than one locus in the rhesus genome were filtered (dataset II). Although the number of genes analyzed was reduced to 1499 in the second dataset, the subset of the genes would be useful in estimating the divergence among the three species. The results were obtained using dataset II in the following manner. The results using the unfiltered dataset (dataset I), which resulted in the inflation of variance, are provided in Additional file 3. Genes that have evolved under positive selection were searched with the model-based likelihood ratio test [40]. In total, 39, 15, and 22 genes showed evidence of positive selection in the human, cynomolgus, and rhesus lineages, respectively (*P *< 0.05). Thirty-eight genes also showed a positive selection signature between the two macaques and 74 were detected in all the three lineages (Table 3). Note that, in Figure 7, the phylogenetic tree is unrooted. The list of positively selected genes is provided in Additional file 4. Excluding the overlapped genes, we identified 101 out of 1499 genes (6.7%) that underwent positive selection in any lineage at 5% significance level. The number of positively selected genes in each of the two macaque lineages was comparable to that estimated in the human-chimpanzee lineages using the same method [41]. Although these candidates of positively selected genes contain many biologically interesting functions, such as transcriptional regulation (*RELA*, *ZNF263*, and *L3MBTL4*), visual perception (*RGS9*, *GPRC5B*, and *RPGRIP1*), and mitochondrial localization (*PET112L*,*VARS*,*ACAA2*,*YARS2*, *FOXRED1*, and *COQ9*) [19], none of the GO categories were statistically overrepresented probably because of the small sample size.

**Table 3:** Number of genes under positive selection out of 1499 non-duplicated genes determined using the branch-site test of positive selection

Lineage^a^	*P *≤ 0.05	*P *≤ 0.01
H-O	39	14
C-O	15	10
R-O	22	21
Between the macaques (C-O + R-O)	37	32
All lineages (H-O + C-O + R-O)	74	33

^a^H: human; C: cynomolgus macaque; R: rhesus macaque; O: cynomolgus-rhesus ancestor (see Fig. 7).

### Genetic divergence between cynomolgus and rhesus macaques

Using the above dataset, we estimated the nucleotide substitution rates of each lineage from the common ancestor of cynomolgus and rhesus macaques (presented in Table 4). Numbers and rates of the non-synonymous and synonymous substitutions for each lineage were estimated using the maximum likelihood method. We assumed that synonymous substitutions are nearly neutral and used them to estimate the divergence time. If we set the divergence of humans and Old World monkeys to 25–35 million years ago (Mya) [42], genes of the two macaques would be considered to diverge 1.9–2.6 Mya on average. However, since the two species diverged very recently, we have to consider the ancestral polymorphisms segregating at the time of speciation [11,12]. Figure 7 illustrates the impact of the ancestral polymorphisms on the estimation of the divergence time. Suppose that the common ancestor of two macaques had the same population size as the population size of extant chimpanzees, *i.e*., 1–2 Mya to the most recent common ancestor [43]. In this situation, only one Mya divergence is assigned to the actual divergence time of the two species. Therefore, without considering ancestral polymorphisms, we tend to overestimate the true species divergence. We applied the maximum likelihood method to estimate the divergence time and ancestral population size of the rhesus and cynomolgus monkeys. As a result, we obtained 2*tu *= 0.00213 ± 0.00022 and 4*N*_*e*_*u *= 0.00327 ± 0.00025 where *t*, *N*_*e*_, and *u *represent the divergence time after speciation, ancestral population size at speciation, and mutation rate with standard errors, respectively. We also noticed that a non-negligible number of nucleotide substitutions were erroneously assigned to the cynomolgus macaque lineage owing to PCR errors in the cDNA libraries. Therefore, the actual substitution rate was estimated by correcting the PCR error using a computational method (see methods). After the correction, we obtained 2*tu *= 0.00181 ± 0.00021 and 4*N*_*e*_*u *= 0.00311 ± 0.00024 (Table 4). If we consider *u *= 10^-9 ^(nucleotide substitution rate per year of Old World monkeys [44]), the divergence time between the two macaques would be estimated as 0.91 ± 0.11 Mya with a standard error. We also estimated the ancestral population size to be 43,200 ± 3300 with a mutation rate per generation of 1.8 × 10^-8 ^in humans [45]. The result suggests that more than a half of the genetic divergence between the two macaques is derived from the ancestral polymorphisms.

**Table 4:** Divergence among the human, cynomolgus, and rhesus genes

**Model without ancestral polymorphisms (Raw data)**		
Lineage^a^	*K*_*a *_(± S.E.)	*K*_*s *_(± S.E.)
H-O	1.06 × 10^-2 ^(3.20 × 10^-4^)	6.82 × 10^-2 ^(1.07 × 10^-3^)
C-O	1.02 × 10^-3 ^(4.79 × 10^-5^)	3.04 × 10^-3 ^(1.20 × 10^-4^)
R-O	4.98 × 10^-4 ^(3.36 × 10^-5^)	2.50 × 10^-3 ^(1.15 × 10^-4^)
		
**Model with ancestral polymorphisms**		
	2*tu*^b^(± S.E.)	4*N*_*e*_*u*^b ^(± S.E.)
Raw data	2.13 × 10^-3 ^(2.24 × 10^-4^)	3.27 × 10^-3 ^(2.52 × 10^-4^)
PCR error corrected	1.81 × 10^-3 ^(2.12 × 10^-4^)	3.11 × 10^-3 ^(2.40 × 10^-4^)

^a^H: human, C: cynomolgus macaque, R: rhesus macaque, O: cynomolgus-rhesus ancestor (see Fig. 7).

^b^*t*: time after speciation; *u*: mutation rate; *N*_*e*_: effective population size of the cynomolgus-rhesus ancestor.

## Discussion

In summary, the sequencing project of cynomolgus monkey cDNAs yielded 85,721 ESTs and 9407 full-length sequences. Since our project mainly studied the brain and testis, the dataset is deficient in other tissue-specific genes, e.g., the genes related to the immune system that many medical researchers would want to explore [46]. The construction of cDNA libraries from other tissues and EST sequencing is still ongoing to complement the transcriptome of cynomolgus monkeys. The latest sequencing status can be confirmed from the website. Because of the close relationship between the cynomolgus and rhesus macaques, cDNA resources of cynomolgus macaques not only are useful for research using cynomolgus macaques but also complement the relative paucity of the transcriptome data from rhesus macaques. Using macaque tissues to scan the primate transcriptome is advantageous because RNA molecules are unstable and are instantly degraded in the tissues during sampling. This causes serious problems for RNA sampling from human tissues, especially in the brain, where fresh samples are rarely obtainable. Therefore, we hope to uncover rare transcripts that would be hidden in the human transcriptome data. In this study, we identified 1024 macaque cDNAs that were not represented in the public human cDNA sequences. Although 51% of the cDNA did not show a positive signal on the microarrays, the following RT-PCR experiments recovered the expression in half (3/6) of the transcripts. The results indicate that these unidentified transcripts were expressed at a low level in the tissues even though the microarray could not detect the expression.

The *M. fascicularis *oligonucleotide microarrays contain probes that matched 8316 known genes and 1024 unidentified transcripts. We determined the number of probe sets for the known genes that overlapped among the commercially available microarray (Affymetrix GeneChip) and previously published microarray of rhesus macaque by Wallace et al., which contains the largest number of probe sets among the published microarrays [47]. Of our 8316 probes for the known genes, 1728 (21%) were not represented in the commercial microarray and 1091 (13%) were not found in the published microarray. Combining the three microarrays, 417 probes for the known genes were represented only in the *M. fascicularis *microarrays [see Additional file 5]. In our preliminary study of the polymorphisms within cynomolgus macaques, we found that the level of polymorphisms in cynomolgus macaques was greater than that in rhesus macaques and slightly smaller than the level of divergence between rhesus and cynomolgus macaques (Osada et al., unpublished data). Therefore, even if we should be careful about sequence mismatches within and between species, the information from both macaque transcripts and the rhesus genome can be combined to build more versatile and comprehensive DNA microarrays that can be used for biomedical surveys using laboratory macaques.

Suppose that we identify positively selected genes in the human lineage after the spilt from chimpanzees. Such genes are useful for understanding the human-specific physiology only when those genes have not been under positive selection in other primate lineages. We identified 37 genes under positive selection between the two macaques at 5% significance level. None of these genes were shared with 387 genes under positive selection in the human or chimpanzee lineages previously determined from the whole genome scan [41], providing support that the method has correctly identified positively selected genes in the specific lineages.

For estimating of the divergence time between cynomolgus and rhesus macaques, we assumed that there is no gene flow between the ancestral species throughout their speciation and divergence time (*i.e*., allopatric model). However, considering the ancestral polymorphisms and the PCR error rate, we estimated the divergence time to be around 0.9 Mya, which is less than the estimation of the age of MRCA of rhesus macaques [10]. Indeed, more than half of the genetic divergence between the two macaques was derived from ancestral polymorphisms. If continuous gene flow is present during speciation, the variance component would be inflated and we would tend to overestimate the amount of ancestral polymorphisms [14].

In this analysis, we used the rhesus macaque genome sequence to represent rhesus macaques. We should note that the rhesus macaque used for genome sequencing was an Indian rhesus macaque; these macaques have genetically differentiated from Chinese rhesus macaques [9]. In addition, our samples of cynomolgus macaques were obtained from different geographic subpopulations. Previous studies using mitochondrial DNA sequences [10] and our preliminary analysis using nuclear DNA sequences (Osada et al., unpublished data) showed that there is a substantial genetic divergence between cynomolgus monkeys of Sundaland (Indonesia and Indochina) and Philippine populations. Therefore, our phylogenetic inference using two sampled sequences has a technical limitation and may be accurate only if there are no complex population structures among the ancestral cynomolgus and rhesus macaque populations. Elucidating the polymorphisms and divergence among macaque species would provide further insight into the evolutionary history of macaques and benefit biomedical research using macaque monkeys.

In Table 4, without correcting the PCR error rate, both the non-synonymous and synonymous divergences are greater in the cynomolgus lineage. This may be due to shorter generation time and smaller population size of cynomolgus monkeys. However, a more reasonable explanation is that the cDNA sequences of cynomolgus monkeys might incorporate the errors resulting from PCR amplification during the construction of the oligo-capped cDNA libraries. The synonymous substitution rate in the cynomolgus lineage is about 0.0005 points higher than that in the rhesus lineage, and the non-synonymous substitution rate differs in about 0.0004 points. Assuming that the selective constraint and generation time of the two macaque lineages are the same, excess divergence of 0.04%-0.05% in the cynomolgus lineage may be an artifact introduced by PCR amplification, which is fairly close to the estimation from the experiment by Suzuki and Sugano [48]. If we reflect the substitution rate in the rhesus lineage to that in the cynomolgus lineage for correcting the errors, the total divergence of the two macaques will be reduced to about 90% (Table 4).

## Conclusion

Transcript data from Old World monkeys provide us with means to determine not only the evolutionary difference between human and non-human primates but also the hidden transcripts in the human genome. Actual cDNA clones of macaques are also indispensable resources for genetic engineering studies. It is considered that the species divergence between rhesus and cynomolgus macaques would be much later than the previous estimates, and the speciation process between them might have been complex. To use laboratory macaques more efficiently, we need to be more aware of the genetic difference within and among macaque monkeys. Increasing the genomic resources and information of macaque monkeys will greatly contribute to the development of evolutionary biology and biomedical sciences.

## Methods

### Cynomolgus monkey samples

Samples from two cynomolgus monkeys, a 16-year-old female (Philippine origin) and a 15-year-old male (Cambodian-Thai hybrid), were used for the cDNA libraries, except for the liver cDNA library (Qlv). The liver samples were collected from three adult cynomolgus monkeys of unknown origin. The monkeys were cared for and handled according to the guidelines established by the Institutional Animal Care and Use Committee of the National Institute of Infectious Diseases (NIID) of Japan and the standard operating procedures for monkeys at the Tsukuba Primate Center, NIID (present National Institute of Biomedical Innovation), Tsukuba, Ibaraki, Japan. Tissues were excised in accordance with all the guidelines in the Laboratory Biosafety Manual, World Health Organization, at the P3 facility for monkeys of the Tsukuba Primate Center. Immediately after collection, the tissues were frozen in liquid nitrogen and used for RNA extraction. Oligo-capped cDNA libraries were constructed according to the method described previously [48]. The prefix in each clone name represents the location of the source of the tissue: Qnp (brain, parietal lobe), Qfl (brain, frontal lobe), Qtr (brain, temporal lobe), Qor (brain, occipital lobe), Qbs (brain stem), Qmo (medulla oblongata), Qcc (cerebellar cortex), Qlv (liver), and Qts (testis).

### Sequencing of cDNA clones

The cDNA clones were sequenced with ABI 3700 and 3730 automated sequencers. The EST sequences were trimmed to avoid the vector sequence of pME18-FL3 [DDBJ/EMBL/GenBank: AB009864]. Entire sequences of the clones were determined by the primer walking method. The repeat sequences at the 5'- and 3'-ends were masked using the Repbase Update database [49] before BLAST search. The BLAST search was performed with an e-60 cut-off value against non-redundant human RefSeq data. The non-redundant data was based on the annotation in the Ensembl Gene database. The longest transcript in the locus was selected as the representative cDNA [24,50]. The macaque cDNA sequences were deposited in the public DNA databases [DDBJ/EMBL/GenBank: CJ430287–CJ493524; BB873801–BB894695; AB303966–AB303967].

### Classification of unidentified transcripts

Classification of the Non-RefSeq transcripts was performed as shown in Figure 1. Transcripts shorter than 300 bp after masking the repetitive sequences were categorized as junk sequences. The remaining sequences were BLAST-searched against all public human cDNA sequences (downloaded on Aug 3, 2007) for the forward strand. Homologous sequences to the human cDNAs were classified as orphan transcripts for the forward strand and anti-transcript for the reverse strand. The remaining 947 clones were mapped on the human genome sequence (build 36.1) by BLAST algorithm and arranged according to the annotation from the UCSC genome browser (hg18). The transcripts that overlapped with the genic regions including UTR were classified as intronic transcripts, and the transcripts that were mapped more than 5 kb away from the genic region were classified as intergenic transcripts.

### Expression assays

Affymetrix GeneChip was designed using the available cDNA sequences of *M. fascicularis*. The chip loads 10,307 probe sets. RNA samples from the cerebrum, cerebellum, liver, and testis of a 3-year-old male cynomolgus monkey were extracted using TRIZOL (Invitrogen) and hybridized to the GeneChip with duplication in a single experiment. The *M. fascicularis *GeneChip contains at most 11 perfect-match probes (25-mers complete matches to the cDNA sequences) and 11 mismatch probes (containing one mismatched oligo) for each probe set, similar to other GeneChip formats. Normalization, signal detection, and signal intensity calculation of the microarrays were performed using Affymetrix MAS5.0 software. Transcripts were considered as expressed when the probe set of both the duplicates agreed for the significant expression (*P *≤ 0.05) [33]. The raw array data were deposited in Gene Expression Omnibus [GEO: GSM201873–201880]. The array design and the sequences of oligonucleotide probes were deposited in the public database [GEO: GPL5396].

### RT-PCR

Templates of the human brain RNA were purchased from Clontech. The macaque brain RNA was obtained from a 21-year-old male cynomolgus monkey. One microgram of total mRNA was amplified using the PrimeSTAR^® ^RT-PCR Kit (TakaraBio). The temperature and time schedules were 30 cycles at 94°C for 20 s, 60°C for 30 s, and 72°C for 1 min. All primer sequences are presented in Additional file 6.

### Human-cynomolgus cDNA sequence alignment

Human-macaque orthologous gene pairs were assigned by the reciprocal best BLAST hit with an e-60 cut-off value. We aligned only that part of the coding sequences (CDS) that was homologous to a BLAST search, because representative human and macaque cDNAs do not necessarily have the same splicing isoforms. The sequences of human and macaque cDNAs were aligned using CLUSTAL W [51], and an unaligned macaque nucleotide was marked by the letter X in the database. Alignments shorter than 100 bp (≤ 33 codons) were filtered for further analysis. In the database, the positions including deletion in the human sequence (or insertion in the macaque sequence) were dropped for estimating the substitution rates. The non-synonymous substitution rate per non-synonymous site (*K*_*a*_) and the synonymous substitution rate per synonymous site (*K*_*s*_) were estimated using the Li-Pamilo-Bianchi method [52,53]. *K*_*a*_/*K*_*s *_ratios were set to 100 in the database when the *K*_*s *_value was zero.

### Human-rhesus-cynomolgus cDNA sequence alignment

The predicted cDNA sequences of rhesus macaques were downloaded from Ensembl (MMUL1.0) and aligned with the human RefSeq sequences. Orthology between the rhesus and cynomolgus genes was confirmed again using the cynomolgus-rhesus reciprocal BLAST hit, and human-rhesus-cynomolgus cDNA alignments were compiled. Alignments containing any frameshifting indels and those shorter than 100 bp (≤ 33 codons) were filtered, which resulted in 2655 alignments (dataset I). The rhesus cDNA sequences were then mapped on the draft genome sequence of the rhesus macaque (rheMac2). The rhesus macaque genes showing > 80% homology to more than one locus on the rhesus genome were removed from the alignments, which yielded 1499 human-rhesus-cynomolgus cDNA alignments (dataset II). To estimate the divergence among the three species, *K*_*a*_(*d*_*n*_) and *K*_*s*_(*d*_*s*_) were estimated using the maximum likelihood method implemented in the PAML program package [54]. We estimated the transition/transversion ratios in 4-fold degenerated sites using the concatenated cynomolgus and rhesus alignments in advance, and fixed the value to the observed value. The test of positive selection was conducted using the branch-site test of positive selection described by Zhang et al. [41], applying the critical values of 2.71 and 5.41 at 5% and 1% significance level without a Bonferroni correction, respectively.

### Estimation of the divergence time between cynomolgus and rhesus macaques

Maximum likelihood estimation (MLE) of the divergence time and ancestral population size was performed using the method of Takahata and Satta [11,14]. MLE was determined using the Newton-Raphson algorithm with many possible initial values. The standard error was determined from the numerically evaluated Fisher information matrix. In order to correct a PCR error rate, we estimated the PCR error rate to be 5.40 × 10^-4^, which was derived from the difference in the synonymous substitution rates of the cynomolgus and rhesus lineages. We assumed that the generation time and the effect of selection on the synonymous sites of the two macaques were the same, and that the erroneous nucleotide incorporated by PCR did not skew. Therefore, when a synonymous substitution in the cynomolgus lineage was found, it was considered that the substitution is because of the PCR error with a probability of 0.178 (5.40 × 10^-4^/3.04 × 10^-3^). We randomly corrected the number of substitutions in the raw data, generated pseudo data for 1000 times, and estimated the evolutionary parameter for each time.

## Abbreviations

CDS, coding sequence; EST, expressed sequence tag; MLE, maximum likelihood estimation; ORF, open reading frame; UTR, untranslated region; MRCA, most recent common ancestor

## Authors' contributions

NO contributed to the designing of the research, performed the experiments and data analysis, and wrote the manuscript. KH, KT, and JK designed the research and contributed to the manuscript. M Hirata performed the computational analysis. YK contributed to the microarray experiments. RT, YU, II, and IT were involved in the cDNA sequencing. M Hida, YS and SS constructed the oligo-capped cDNA libraries. All authors read and approved the final manuscript.

## Supplementary Material

Additional file 1 Expression of novel macaque transcripts. Significance of gene expression in the *M. fascicularis *oligonucleotide microarray analysis is shown. Click here for file

Additional file 2 Unidentified UTR regions in the macaque cDNAs. The regions of macaque cDNAs that did not show homology to human cDNAs are listed. Click here for file

Additional file 3 Divergence among the human, cynomolgus, and rhesus genes (dataset I: without a duplication filtering). Estimation of gene divergence using 2655 human-rhesus-cynomolgus alignment is shown. Click here for file

Additional file 4 LRT (likelihood ratio test) statistics for the test of positive selection. Candidate genes under positive selection using branch-site test of positive selection are given with log-likelihood ratio. Click here for file

Additional file 5 Specific probes for known genes in the *M. fascicularis *microarray. These genes are not found in the previously published macaque oligonucleotide microarray. Click here for file

Additional file 6 Primer sequences for RT-PCR. The list shows the primer sequences that were used for RT-PCR. Click here for file
