# Supplementary material for: Large-scale analysis of Macaca fascicularis transcripts and inference of genetic divergence between M. fascicularis and M. mulatta
Source: BMC Genomics. 2008 Feb 24;9:90. doi: 10.1186/1471-2164-9-90 (PMC2287170; doi:10.1186/1471-2164-9-90)
Supplement: Additional file 3 — Divergence among the human, cynomolgus, and rhesus genes (dataset I: without a duplication filtering). Estimation of gene divergence using 2655 human-rhesus-cynomolgus alignment is shown. [file 1471-2164-9-90-S3.doc]

Divergence among the human, cynomolgus, and rhesus genes (dataset I: without a duplication filtering).

| **Model without ancestral polymorphisms (Raw data)** | | |
| --- | --- | --- |
| Branch label* | *K_a_* (± S.E.) | *K_s_* (± S.E.) |
| H-O | 9.81 × 10^-3^ (2.49 × 10^-4^) | 6.74 × 10^-2^ (8.27 × 10^-4^) |
| C-O | 1.21 × 10^-3^ (7.23 × 10^-5^) | 3.58 × 10^-3^ (1.39 × 10^-4^) |
| R-O | 9.76 × 10^-4^ (1.00 × 10^-4^) | 3.25 × 10^-3^ (1.71 × 10^-4^) |
|  |  |  |
| **Model with ancestral polymorphisms** | | |
|  | 2*tu* (± S.E.) | 4*N_e_u* (± S.E.) |
| Raw data | 1.18 × 10^-3^ (1.43 × 10^-4^) | 5.83 × 10^-3^ (2.03 × 10^-4^) |

* H: human, C: cynomolgus macaque, R: rhesus macaque,O: cynomolgus-rhesus ancestor (see Fig. 2).
